# Improving the quality of life of children and parents with nocturnal enuresis: the role of health education

**DOI:** 10.3389/fped.2024.1464465

**Published:** 2024-12-24

**Authors:** Jianrong Liao, Lin Zhu, Danfeng Xie, Xiaomeng Wang, Ping Zhou

**Affiliations:** ^1^ Department of Pediatric Nephrology and Rheumatology, Sichuan Provincial Maternity and Child Health Care Hospital, Chengdu, Sichuan, China; ^2^ School of Nursing, Chengdu Medical College, Chengdu, Sichuan, China; ^3^ Department of Pediatric Surgery and the Pediatric Nephrology Laboratory, Sichuan Clinical Research Center for Pediatric Nephrology, Chengdu, Sichuan, China

**Keywords:** nocturnal enuresis, quality of life, health education, children, parents

## Abstract

The sleep disorder nocturnal enuresis (NE) affects children's health and quality of life, as well as places a heavy burden on their families. Treatment improves the child's quality of life. Unfortunately, some parents do not seek treatment for their children because they are unaware or misinformed about NE. The goal of health education is to enhance or maintain the patient's health status, thereby enhancing the patient's quality of life through positive coping strategies. Educating children and parents about NE is an effective way for nurses to raise their awareness. Furthermore, they can improve the quality of life of children by promoting their active participation in treatment. The purpose of this paper is to review information about NE and explore the role of health education in improving children's and parents' quality of life.

## 1 Introduction

International Childhood Continence Society (ICCS) defines nocturnal enuresis (NE) as involuntary urination during sleep that lasts at least three months and occurs twice a week (1). NE is a common condition in childhood (2). According to studies, NE prevalence ranges from 4% to 18% (3). It is possible for different countries or regions to have different prevalence rates due to differences in definitions and factors such as geography, environment, health resources, culture, and education. The prevalence of NE in China ranges from 4.07% to 10.03% (4). It is more common among boys before puberty, while it is equally prevalent among girls after puberty (1, 5).

NE is caused by various factors, including genetics, increased nocturnal urine output, reduced bladder capacity or urethral sphincter dysfunction, and sleep-wake disturbances (4, 6). Among them, sleep disorders, especially sleep apnea disorders, interact with NE (7–9). NE increases with the prevalence of OSA, and children with NE are more likely to develop OSA than normal children. And the symptoms of some children with NE may improve after treatment of OSA (9). OSA causes nocturnal hypoxia and carbon dioxide retention, which in turn causes a series of physiological changes, such as sympathetic arousal, elevated blood pressure, and increased cardiac load. These physiological changes may affect blood perfusion and urine production in the kidneys, leading to increased nocturnal urine output and thus increasing the risk of NE. In addition, frequent apneas and awakenings can result in sleep fragmentation, making it challenging for children to enter the deep sleep stage of sleep structure disruption, which in turn interferes with the normal function of the bladder, leading to the development of NE (7). Numerous studies have also suggested that excessive psychological pressure and family environment contribute to the development of NE (5). Children who have NE are less likely to develop NE as they get older. Children with NE recover spontaneously in about 10%–15% of cases (10). Chronic bedwetting, however, can negatively affect children's social and psychological well-being (11). Several studies have shown that children with NE have a lower quality of life than those without it (11, 12). In addition, parents of children with NE may experience increased psychological pressure and caregiving burden as a result of frequently changing and washing urine-soaked bedding and clothing (13).

The lack of knowledge and attention about NE, however, leads to some parents not seeking medical consultation or treatment for their children. The survey results show that only a portion of parents, approximately ranging from 2.1% to 55%, will seek treatment for their children (14). Enuresis education, including its causes and effective treatments, has been shown to change parents' views and misconceptions about the condition. This can increase attendance rates at health appointments, and enhance the quality of life for both children and parents (15).

Health education plays a vital role in improving the quality of life for children and parents in NE. Through health education, parents can be informed of NE's negative effects on their children. Treatment is an effective way of dealing with NE. Caldwell et al. Showed that children's quality of life could be boosted by effective treatment (16). At the same time, health education can also enhance children's self-confidence and alleviate anxiety and low self-esteem caused by bedwetting (15). Therefore, providing relevant information on NE is critical for improving children's psychological well-being and quality of life.

Several previous studies have emphasized the importance of correcting families' misconceptions, raising their awareness, and ensuring timely medical attention to improve children's and families' quality of life (4, 14, 17). However, current research focuses on exploring treatment modalities, epidemiology, and pathogenic mechanisms for NE (18). Specific educational methods and strategies are still not fully explored. In this review, we summarize the existing knowledge about NE and to examine the potential benefits of health education for children and families affected by NE. The aim is to provide useful clinical information to children and families affected by NE and to enhance their quality of life.

### 1.1 The impact of NE: on children and families

Numerous studies have found that children and families affected by NE have a significantly lower quality of life than children without the condition (1, 12). Mohamed et al. reported that NE is common and distressing for children and their families (19). Among its symptoms are shame upon awakening, fear of being discovered by friends, and psychological stress caused by parental punishment. In Iscan et al.'s study, it was found that children with enuresis had a poorer quality of life. Among the quality of life domains assessed, self-esteem, emotional well-being, and relationships with family and friends were the most vulnerable (20). In a similar study on the quality of life of children with NE, it was noted that children with NE are prone to low self-esteem, shame and guilt, and fear of being discovered, ridiculed (21). In addition, NE can also lead to sleep disorders, resulting in sleep deprivation at night. Children with NE may experience difficulty concentrating in the classroom, which can result in low grades (22).

It has a serious impact on the social lives of children who suffer from NE. Surveys indicate that bedwetting is the third most disruptive factor in children with NE, behind divorce and parental conflict (1, 23). In a study on children's psychological anxiety and depression, Ylmaz et al. found that bedwetting negatively affects academic performance and interpersonal relationships (24). A study by Eray et al. reported that children with NE tend to feel uneasy and avoid social situations when entering a new environment (25).

As a result of economic development and improved literacy, parents and medical professionals are becoming increasingly concerned about NE (4). Some parents also realize that enuresis is out of control. However, several studies have pointed out that if the child continues to wet the bed with age, parents do not condone it (12, 26). They often blame and penalize the child. Al-Zaben et al. noted that children who were punished for bedwetting had more severe NE than children who were not punished for enuresis, and that children who were punished for frequent and prolonged bodily injury had more severe depressive symptoms and poorer quality of life (27). A survey on NE in Italy found that 54.1% of parents punished their children for bedwetting (28).

NE affects the entire family, not just the child. A long period of changing bed covers and clothes after bedwetting can be exhausting and frustrating for parents. Arguing between parents about who changes the sheets and clothes can be stressful for family members. Tension also adversely affects physical and mental development (3, 17). At the same time, mothers caring for a child with NE may experience a decrease in their quality of life. They may experience feelings of anxiety, stress, and helplessness (26). In addition, families suffering from NE often spend more on laundry, bedding, disposable diapers, and mattress replacements (29).

## 2 Factors affecting the quality of life

### 2.1 Children's factors

Study found that children with NE have a quality of life influenced by gender and age (20, 30). Michel et al. observed that quality of life scores decreased with age, and girls scored lower than boys (31). Kilicoglu and Van et al. came to similar conclusions (32, 33). In addition, some studies have found older children also report more psychosocial problems, particularly regarding their family and self-concept of appearance (2). There are several possible reasons why this may be the case. First of all, their cognitive level and self-care ability gradually increase with age, which makes them more afraid of being discovered by their friends for bedwetting, and they show lower self-esteem, shame, and exclusion from social activities (34). In addition, it may be related to cultural background. In some countries, especially in Asia, parents tend to pay more attention to boys, believing they will inherit the family's power and duties and have a greater responsibility to support them. Because of this, parents are more willing to spend money, time, and resources on boys than on girls (31).

### 2.2 Family factors

Several factors affect the quality of life of children with NE, including caregiver literacy (28, 35). Research suggests that children from families with lower education levels may be at higher risk of enuresis (36). Furthermore, studies have found that mothers are typically the primary caregivers of children with NE, and their level of education can influence their management style (37). Mothers with higher levels of education are more likely to recognize abnormalities in their children and actively seek medical attention, compared to mothers with lower levels of education. Schlomer et al. found that caregivers with postgraduate education were more likely to seek healthcare for their children (38). The well-being of children with NE is also affected by parents' perceptions and attitudes (2). Less educated parents may have misconceptions about NE, believing that bedwetting is a problem of self-control. Therefore, when bedwetting persists for a long period, they may perceive it as a failure of their education. They may experience anxiety, guilt, and loss of confidence. Finally, they may even blame and scold their children (39).

There is a correlation between economic income and treatment success (24). Families with lower incomes have a higher prevalence of NE, according to studies (3, 28). Children from low-income families with enuresis are at risk of low quality of life (20). Treatment with NE may not be effective in the short term and requires long-term adherence. Thus, the cost of treatment may place more of a burden on less well-off families (17). In addition to treatment costs, managing the child's daily needs will also increase the family's financial burden compared to a normal child.

## 3 Health education approaches

According to the survey, many parents and children are unaware of NE. To ensure that parents and children understand and accept NE treatment, medical professionals must disseminate information about the disease in a health education setting. It will reduce the impact of NE on children and their families, improving their overall quality of life in the long run (16, 24). In the medical system, health education is crucial in improving patients' health literacy, promoting disease recovery, and preventing disease recurrence (40). Traditional health education is delivered through face-to-face teaching, distribution of health education manuals, telephone counseling, or outpatient follow-up visits. As technology advances and medical paradigms change, forms of health education are evolving. For example, virtual reality technology and the internet can now be used together (41, 42).

By integrating modern technologies into health education, we can enhance the effectiveness of health education and thereby improve the satisfaction of our patients (43). Regardless of the form of health education, the most important thing is to convey medical information and knowledge accurately. In order to achieve this goal, nurses must understand the advantages and disadvantages of each type of health education (see Table 1) and how best to communicate disease information to patients and their families.

**Table 1:** Advantages and drawbacks of forms of health education and their applicable setting.

Forms of health education	Advantages	Drawbacks	Applicable setting
Face-to-face education	Receive real-time patient feedback. Personalized answers to patients’ questions. It facilitates the building of trust and emotional connection and enhances patient treatment compliance.	High cost of time spent. There are time and space constraints. Information dissemination is limited in speed and scope.	Seniors. Patients with chronic diseases. High-risk groups (heart attack patients, pregnant women, etc.). Children and adolescents.
Paper-based brochures	Ease of dissemination and preservation. Reusable. Comprehensive content.	High production costs. Untimely updating of information. Limited audience reach.	Seniors. Patients with chronic diseases.
Internet and health education	Wide coverage, no geographical limitations. Convenient and efficient, not limited by time and space. Information is updated in real-time. Variety of formats, including graphic, audio, video, etc. Resource consolidation can be achieved.	The quality of information varies. Privacy and security issues. There is a certain technical threshold.	Young population. Patients with chronic diseases.
Virtual reality (VR) and augmented reality (AR)	Immersive experience to deepen understanding and memory. Interactivity. Visual education to visualize some of the scenarios.	High equipment costs. High technical requirements. May bring about adverse reactions such as dizziness and discomfort.	Patients with motor and cognitive disorders requiring rehabilitation. Post-operative recovery training. Patients with chronic disease management, pain management, and psychological governance. Children and adolescents.

Face-to-face health education is part of the traditional health education approach (43). Direct face-to-face communication and interaction with the patient are used to convey information about the disease. It is a very popular method of health education. However, in the process of face-to-face health education, some patients and their families are prone to problems such as incomplete information acceptance, insufficient information processing ability, and incorrect information memorization (44). As a result, healthcare professionals may need to inform patients multiple times to achieve an educational effect, resulting in increased time and costs.

A health education manual is a traditional form of health education. The document is comprehensive and can be kept for a long time. Medical terminology, however, may be difficult to understand for some patients and their families (19). In order to assist patients and families in understanding their diseases, healthcare providers can use visual aids, such as pictures.

Globally, 65.6% of the population has access to the Internet (45). As a result, the Internet is a highly beneficial tool for health education. The use of the Internet to facilitate health education helps patients transcend the limitations of time and distance. Online information can be accessed anytime and reviewed multiple times as desired. There is, however, a lack of quality and variability in health information currently available on the internet (46). There may be some patients who have difficulty screening information and are therefore more likely to misjudge false information.

Virtual reality (VR) and augmented reality (AR) are emerging technologies. Technologies like these are used in gaming and entertainment. However, researchers are now also exploring their potential in medical applications, such as critical care medicine, rehabilitation, and pediatrics (41, 47). VR enables users to immerse themselves in a three-dimensional virtual world, while AR allows digital information to be superimposed onto the real world (47). VR and AR offer unique immersion, presence, and interactivity. They can make health education content more interesting and engaging, and help patients and families understand abstract medical knowledge (48–50). It is likely that verbal preaching will not be effective with children due to their limited understanding. VR and AR, however, create a sense of immersion that children can relate to. This makes it easier for them to understand the disease and also reduces the anxiety caused by the disease. VR and AR are effective tools for delivering disease-related information, helping patients understand their disease, and improving their compliance with treatment. There is, however, a limit to the adoption of VR and AR technologies due to their cost, the need for professional equipment, and the lack of technical support.

Researchers in Egypt have observed that implementing a health education learning package can prove to be an effective way for mothers of NE children to improve their knowledge, practices, and attitudes, while at the same time promoting their child's compliance with treatment. However, the health education learning package was based on pre-existing questionnaires designed specifically for the mothers involved. Additionally, the health education learning package employs a variety of health education methods and has been reviewed repeatedly by experts. Despite the high quality of the content, the design and implementation of the health education learning package is time-consuming (16). Video content about enuresis was analyzed on YouTube by Toprak et al. Video quality appears to vary widely based on their findings. In spite of the fact that some content may be helpful to patients and families facing difficulties, the presence of low-quality videos may mislead them (42). Health education for children with NE is currently disseminated through mass media, posters, brochures, maternal and child health centers, and individual counseling. Virtual reality technology has not been studied in children with NE, but it may be explored in the future.

## 4 Implementation strategies for health education

As part of the treatment process for NE, health education plays a complementary but crucial role. To improve compliance and ensure treatment success, children and parents need to have a basic understanding of the principles and methods of treatment before treatment begins, whether medication or behavioral therapy. Meanwhile, healthcare professionals must answer promptly to any problems encountered during treatment. It is well known that health education can be very helpful when it comes to improving treatment and quality of life. Thus, NE treatment methods should be integrated with health education to optimize outcomes and improve compliance.

### 4.1 Behavioral interventions

Behavioral interventions are an initial approach to treating NE. It includes adjusting habitual routines, developing appropriate urination and defecation habits, implementing reward mechanisms, and keeping a urine loss diary (4, 51). These measures can be used alone or in combination with medication or alarms.

One of the etiological factors of NE is a nocturnal reduction in bladder capacity or overactivity of the urethra (1). Therefore, several studies have suggested appropriate solutions to this etiology. Firstly, children drink normally during the day, drinking their daily amount of water. At night they limit their water intake, avoiding all liquids, especially two hours before bedtime. Secondly, empty your bladder before heading to bed. Thirdly, foods or drinks containing theophylline and caffeine can irritate the bladder, so they should be avoided in children, especially at night. Finally, excessive salt intake can lead to increased nocturia, so salt intake should be controlled during dinner (6, 52, 53).

It is possible to treat NE by developing healthy urination and defecation habits (29). It has been found that excess feces in the colon can reduce bladder capacity and that relieving constipation reduces NE (54). To maintain clear stool, children should consume more foods with crude fiber and have regular bowel movements. Children with constipation should also be treated aggressively to avoid interference with NE (55).

Some parents are unaware of enuresis and blame and punish their children for frequent bedwetting. But the truth is that punishment and chastisement to cure bedwetting are counterproductive (6). Therefore, it is very important for healthcare professionals to provide parents with information about the pathogenesis of NE so that they understand that bedwetting is not within the child's control. In addition, studies have shown that encouragement reduces psychological distress and improves treatment adherence (56). Therefore, parents can provide appropriate encouragement to help their children realize that bedwetting is not their fault and reduce psychological pressure. In addition, during the treatment period, parents can reinforce the child's positive behavior and motivation for treatment. This can help the child actively participate in treatment and improve therapy success rates.

It is imperative to keep a urination diary. It is noninvasive and effective, and can objectively assess the child's condition, determine the type of NE, and guide follow-up treatments (57). Additionally, it can help evaluate treatment compliance by the child and family and supervise treatment more effectively. Kyung et al. observed that questionnaires may be subject to memory bias and subjectivity, whereas urinary diary records provide more objective and reliable information (58).

Behavioral interventions play an active role in the treatment of children with enuresis as a complement to first-line treatment. Due to its low cost and lack of risk, behavioral therapy is often preferred by parents who worry about medications' potential negative effects on their children (59).

### 4.2 Alarm therapy

**Figure 1:**
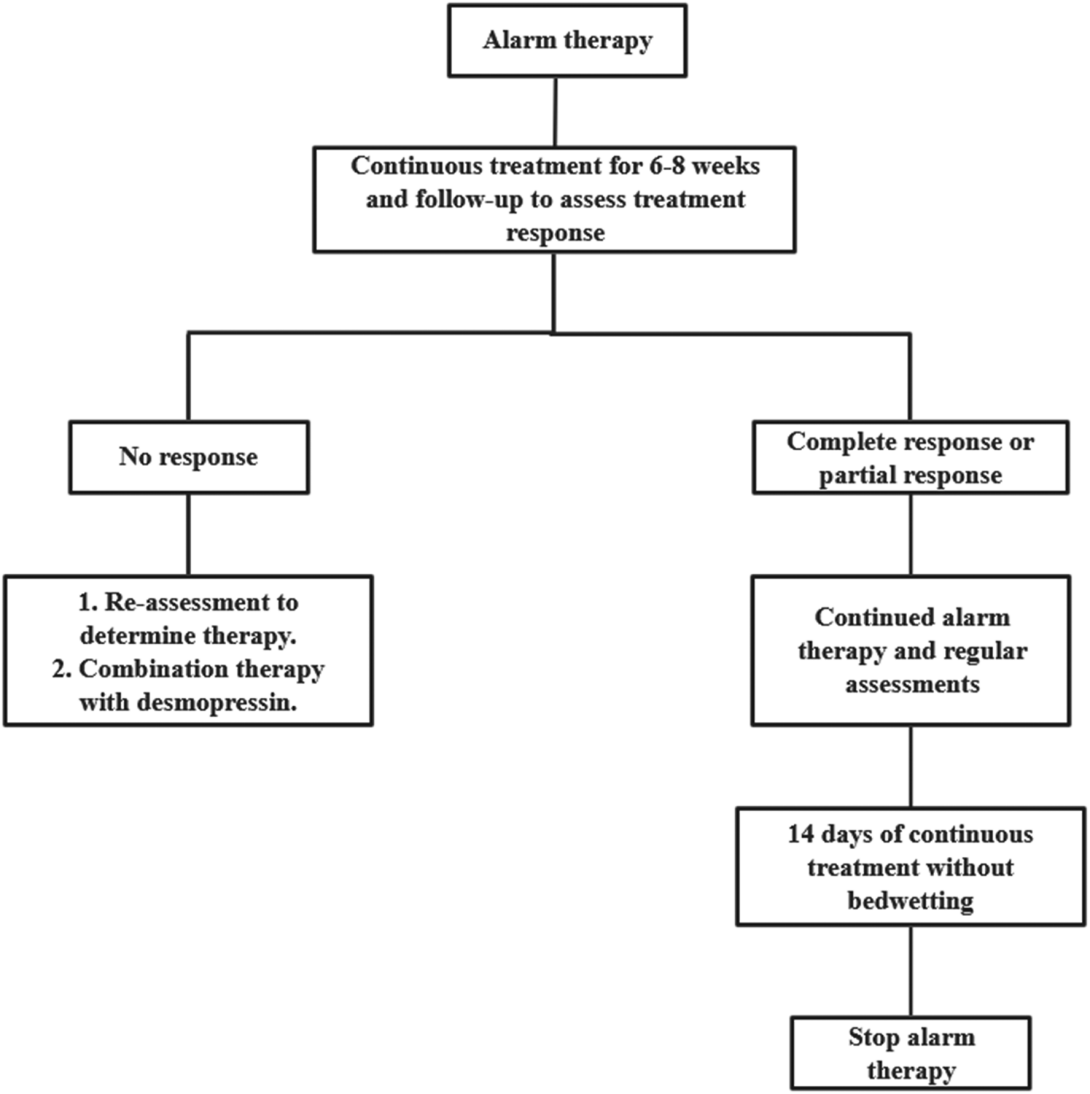
Alarm therapy treatment process. By consensus, an expert committee developed a protocol for evaluating and treating pediatric nocturnal enuresis patients in mainland China. No response: 50% reduction in bedwetting frequency; Partial response: 50% to 99% reduction in bedwetting frequency; Complete response: 100% reduction in bedwetting frequency.

Enuresis alarms are considered one of the first lines of treatment for enuresis (16). It is an electronic device that consists of an alarm and a humidity sensor. There are two main types: body-worn and padded, while the fixed-on-the-body alarm type is the choice for more people (52). The alarm works on the principle that once the humidity sensor attached to the trousers is activated, it will wake up the child via an alarm like a ringing bell (60). This treatment assists children in developing a regular mechanism for urinary arousal during sleep. This is done by enhancing their ability to store urine at night and reducing nocturnal urination. Based on the survey, the success rate of treating enuresis using alarms ranges from 62% to 70% (52). Figure 1 illustrates the alarm therapy treatment process (57).

Even though alarms can effectively treat NE, there are some issues with the treatment process. Alarms can be time-consuming and exhausting for both children and their families (61). According to the ICCS, parents should stay with their child while the alarm sounds, allowing the child to turn off the alarm and go to the bathroom to urinate, thus maximizing the therapeutic effect (60). However, a prolonged alarm can disrupt parents' and children's sleep, resulting in fatigue during the day. Alarm therapy can be interrupted as often as 30% according to a survey (62). Alarm treatment effectiveness can only be determined after at least 6 to 8 weeks of continuous treatment (10). Therefore, if children and parents do not see results in the treatment process, they may lose motivation and discontinue treatment.

Alarms are not suitable for all NE children. Parents and children who are motivated to follow the treatment may be more effective with alarms when bedwetting episodes are regular and urine output exceeds 65% of the expected volume. A better option might be medication for children with NE whose parents don't support them, who are stressed, or who lack motivation (29, 52).

Health education is recommended before alarm therapy to reduce dropout rates and enhance treatment efficiency. For instance, the nurse should illustrate the alarm's operation to ensure that both parents and children comprehend its functionality. Nurses should also advise parents to stay with the child as much as possible during night-time treatments to help the child develop effective treatment habits and achieve therapeutic results. Furthermore, it is essential that parents and children are made aware that the alarm treatment is a process that necessitates time and patience. This is to ensure that they are psychologically prepared for the long-term nature of the treatment, thus preventing them from abandoning it prematurely due to the lack of immediate visible outcomes. Additionally, it is crucial for parents and children to comprehend the efficacy of the treatment approach, which will in turn bolster their confidence in the treatment. During treatment, parents can explain the effects of enuresis and the importance of treatment in a way that the child can understand, so that the child understands why treatment with an alarm is necessary. Parents should encourage their children to play an active role in the treatment process, thereby fostering a sense of responsibility and self-confidence. Concurrently, parents must also be mindful of their children's emotional fluctuations and provide timely encouragement and support when their children encounter difficulties or setbacks.

### 4.3 Medication

#### 4.3.1 Desmopress

**Figure 2:**
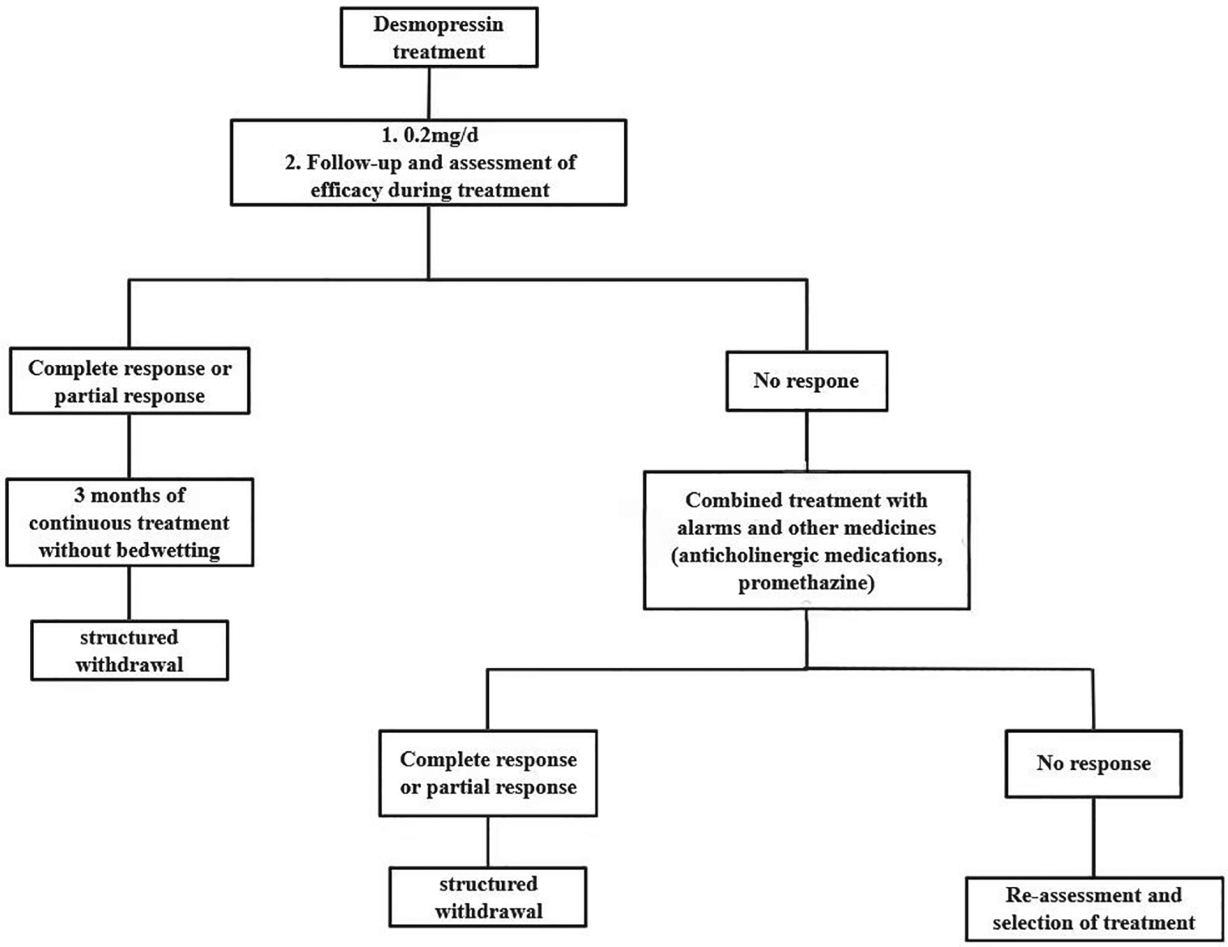
Desmopressin therapy treatment process.

Desmopressin is another effective option for treating NE. For NE, it is considered one of the first-line drugs (63). Desmopressin is a synthetic analog of an anti-diuretic hormone. It reduces nocturnal urine production by increasing water reabsorption in the renal tubules and regulating renal ion secretion (64). In this way, it is more effective at treating NE caused by antidiuretic hormone deficiency. A complete response is achieved by 20%–30% of patients receiving desmopressin treatment, whereas a partial response is achieved by 20%–40% of patients (65). Figure 2 illustrates how desmopressin therapy works (57).

Desmopressin can be administered in different ways, such as nasal sprays, nasal drops, oral tablet capsules, and frozentablets (66). Hyponatremia and seizures have been reported following the transnasal desmopressin administration. The United States and some European countries have therefore banned its use (66, 67). A study by Keten et al. noted that freeze-dried desmopressin tablets have a higher success rate than other forms of the drug (63).

Desmopressin should be taken one hour before bedtime or two hours after dinner, while also limiting fluid intake at night to prevent hyponatremia (29, 52, 60). To begin with, Desmopressin should be given at a low dose of 0.1 to 0.2 mg. The dosage could then be adjusted based on the therapeutic effect. Two to four weeks after starting the treatment, it is recommended to evaluate its effectiveness. If the treatment is effective, the dose can be maintained and continued for at least 3 months (52, 66).

Despite being faster-acting than alarm therapy, desmopressin treatment has a significant disadvantage: a high relapse rate. After 12 weeks of desmopressin treatment, the relapse rate was 50% compared to only 12% with alarm therapy (63). A structured discontinuation method, i.e., discontinuing desmopressin in a stepwise manner, has been suggested to reduce the relapse rate (68, 69). However, some studies suggest that there is no difference between the two methods (direct cessation or structured withdrawal) in terms of reducing recurrence rates (69).

The nurse reminds parents to administer medications to their children strictly in accordance with doctors' orders to ensure that children take the correct dose of medication at the correct time. At the same time, supervise children to limit their fluid intake at night and develop good living habits. It is also necessary to teach parents to closely monitor the physical condition of their children during the medication period. If any related complications or abnormalities occur, they should contact health professionals in time. In addition, nurses need to follow up with parents and children on a regular basis to see how the treatment is progressing and to assess the efficacy of the medication and the child's physical response.

#### 4.3.2 Anticholinergic medications

Anticholinergic medications such as tolterodine and oxybutynin can also treat NE, in addition to desmopressin medications. It primarily works by relaxing the bladder muscles, increasing bladder capacity, and improving the overactive or unstable state of the urethral muscles (70). This reduces the frequency of urination and enuresis. Consequently, they are more effective in children with reduced bladder capacity or overactive bladders. When urine loss alarms and desmopressin do not work, anticholinergic drugs are usually used. However, anticholinergic medications are not effective alone, so they are usually combined with desmopressin (52). Some studies have shown that the combination of desmopressin and oxybutynin significantly increases the number of patients with complete and partial responses (71, 72). Additionally, anticholinergic drugs have several potential side effects, including dry mouth, dry eyes, constipation, headaches, and urinary retention (73, 74). It is therefore critical to use them carefully and cautiously.

#### 4.3.3 Imipramine

Imipramine is a tricyclic antidepressant that can also treat NE. The mechanism by which it treats NE is unknown. It has been shown that these drugs stimulate adrenergic receptors, modify sleep patterns, and decrease excessive night-time sleep. As a result, patients can wake up once their bladder is full. They also inhibit acetylcholine, which affects bladder contraction and urethral sphincter relaxation. Reducing the frequency of involuntary bladder contractions and increasing urine storage capacity, can help patients improve their nocturnal bedwetting symptoms (56, 75). This treatment benefits 30%–50% of patients with NE (76). Due to its potential cardiotoxicity, imipramine should be used cautiously when treating NE. An overdose can cause cardiac conduction disturbances and myocardial depression (76, 77). In addition, tricyclic antidepressants may be associated with adverse effects such as dry mouth, constipation, blurred vision, tachycardia, weight gain, headaches and dizziness, behavioral changes, irritability, and drowsiness. So for safety reasons, it has been used as a third-line treatment option (77). However, imipramine is relatively inexpensive compared to other treatments, so it is an optional treatment for families with low incomes (52).

### 4.4 Chinese medical treatment

Recent years have seen an increase in the use of traditional Chinese medicine (TCM) for NE clinical treatment (78). In NE treatment, TCM is effective without significant side effects. Treatments include herbal medicine, acupuncture, and tuina (78, 79).

Suoquan Capsule is a herbal medicine commonly used to treat NE. It contains Radix Linderae, Alpinia oxyphylla, and yam, which reduce urethral muscle excitability and concentrate urine. This makes it an effective treatment for NE. In the study by Ma et al, shrinkage capsules along with desmopressin were more effective and had a lower relapse rate than shrinkage capsules alone (79).

Acupuncture stimulates specific points such as Guanyuan, Sanyinjiao, Ashigaru, and Bladder Yu, improving nerve function and bladder control (80). Enuresis can be treated with laser acupuncture, which is less invasive and painful than traditional acupuncture (81). Studies have shown that laser acupuncture is a painless and non-invasive treatment with a low recurrence rate and no side effects. In some cases, it can be considered a viable alternative to NE (82, 83). There is controversy regarding whether laser acupuncture is more effective in treatment than desmopressin. The effectiveness of laser acupuncture and desmopressin for the treatment of enuresis in children was compared by Radmayr et al. (2001). In the study, forty children were randomly divided into two groups, one receiving desmopressin and the other receiving laser acupuncture. Neither modality had a significant difference in treatment success (84). However, in a similar study, Alsharnoubi et al. (2017) found that laser acupuncture was statistically significantly more effective than desmopressin in a similar study (85).

A common treatment for NE in China is pediatric tuina (86). It is similar to the mechanism of action of acupuncture, in specific parts of the body surface, the use of chiropractic, kneading, massage, and other techniques to give a certain amount of stimulation, which increases qi and blood flow and enhances bladder function. The effects of tuina have been shown to affect signaling in various brain reflex zones (86, 87). Treatment for enuresis using tuina can be a non-invasive, painless, and side-effect-free alternative.

TCM treatment has fewer side effects than Western medical therapies, even though there is not enough evidence to conclude that TCM is more effective than Western medicine. Thus, TCM can be considered as an alternative treatment option for enuresis. Before treatment, nurses should explain to parents the specific characteristics, applicable conditions and possible effects of traditional Chinese medicine therapies such as herbal medicine, acupuncture and tuina, and help parents to choose appropriate Chinese therapies or integrative therapies according to their children's specific conditions, such as age, severity of symptoms, physical tolerance, and so on. All therapies require a certain course of treatment to be effective. It is the responsibility of parents to encourage their children to engage actively with the treatment, while simultaneously maintaining a record of their child's progress and any changes in symptomsso that they can provide timely feedback to the doctor.

## 5 Discussions

Behavioral interventions are an initial approach to treating NE, and throughout the course of treatment (51). Some parents may lack an understanding of the necessity of behavioural interventions and are unable to effectively supervise their children in order to implement them, which affects the treatment outcome. Nurses assist parents and children in understanding the principles and importance of behavioural interventions through the provision of health education, thereby increasing the likelihood of parental and child cooperation with changes in lifestyle habits and active participation in treatment.

Although alarm therapy for NE has been demonstrated to be highly effective, it also has a high rate of interruption (52, 62). If the child and parents are not aware of the long-term nature of the treatment and the potential issues that may arise, such as disrupted sleep during treatment and the lack of immediate improvement, they may lose confidence and terminate the treatment prematurely, thereby preventing the treatment from achieving its intended outcomes. Furthermore, a lack of comprehension regarding the correct utilisation of the alarm may result in suboptimal treatment outcomes. Therefore, nurses need to explain to parents and children how the alarm works and how to use it before the treatment is given to ensure that they can use it properly. Parents and children should also be made to understand that alarm treatment is a process that requires time and patience, so that they will not easily give up the treatment because they do not see any results in the short term or encounter difficulties.

It is important to note that pharmacological treatment is not without side effects, and desmopressin medication is also associated with a high relapse rate (63, 73, 74, 77). Consequently, when selecting a medication to treat NE, parents are apprehensive about the potential adverse effects on their children and may be reluctant to cooperate. Through health education, nurses provide parents with comprehensive information about the principles of drug treatment, including the timing of administration, dosage, and potential adverse effects. This enables parents to make informed decisions and enhances their cooperation with the doctor's treatment plan, ultimately improving the adherence and effectiveness of the treatment.

Some parents may have misconceptions about TCM treatments, thinking that their effects are unclear or not believing that they can cure NE. The nurse explains to the parents the specific characteristics, application and possible effects of TCM treatments such as Chinese herbs, acupuncture and tuina (78), helping them to choose the appropriate treatment according to their child's specific condition. At the same time, parents and children should be informed that the side effects of TCM are relatively few, so as to increase their compliance with treatment.

As technology advances, treatments are being updated. From the literature, it has been found that a new product is now being developed for the treatment of NE, a safe, comfortable, non-invasive pre-voidance wearable alarm that measures bladder filling using ultrasonic sensors to trigger an alarm prior to urination (88–91). Traditional alarms can only react to a bedwetting event after it has occurred and cannot give advance warning (60). The pre-voidance alarm is more proactive and preventive as it can alert the child before he/she is about to urinate, giving him/her enough time to wake up and go to the toilet to urinate. And it can also adjust the threshold and timing of the alarm trigger to suit each child's specific needs through multiple measurements and learning from the bladder (90). In conclusion, by triggering the alert at the pre-urination stage, it helps to develop bladder control, and over time, the child may gradually learn to control his/her urination behaviour, reducing the frequency of nocturnal enuresis and improving the therapeutic outcome. At the same time, warning the child before bedwetting gives him/her the opportunity to control urination on his/her own, avoiding the occurrence of bedwetting incidents and helping to protect the child's self-esteem (91).

This study covers a variety of treatments for enuresis, including behavioural interventions, alarm therapy, multiple medications, and TCM, and elaborates on the importance and specific content of health education in each of these treatments. This provides a more comprehensive guide to clinical practice, enabling healthcare professionals to carry out health education effectively at different stages of treatment according to the conditions of different patients. With the development of technology, this study also explored the potential of new technologies such as VR and AR in health education. Although they are not yet widely used in health education for the treatment of enuresis, it is suggested that these technologies can make the content of health education more interesting and easier for children to understand, providing new directions and ideas for future research and practice.

## 6 Conclusion

Nocturnal enuresis (NE) is one of the most common chronic disorders of childhood, affecting children's health and quality of life, as well as placing a heavy burden on families. Even though some children may recover with age, most may experience long-term effects if they do not receive treatment. Health education plays a vital role in improving the quality of life of NE children and parents. Nurses communicated information about NE to children and parents through health education, which increased their awareness of NE and reduced their anxiety and worry. At the same time, this study summarises the treatment approaches for NE and the role of health education, which can inform healthcare professionals when developing treatment plans and providing patient education. Secondly, this study also highlights the need for further research into more effective health education models and strategies, especially in the context of emerging technologies such as virtual reality and augmented reality. Future research could focus on filling the research gaps identified in this study, such as exploring the long-term effectiveness of different treatments in combination with health education, as well as investigating how to better tailor health education to the individual characteristics of patients and families. In conclusion, this study contributes to a deeper understanding of NE and the importance of its health education. It provides valuable insights for improving the quality of life of children with NE and their parents.

## References

[1] Huang W Liang Y Yang Q Ma X. Nocturnal enuresis in children: parents’ perspectives. Nurs Open. (2022) 9(5):2335–41. 10.1002/nop2.1243 35661439 PMC9374395

[2] Tai TT Tai BT Chang Y Huang K. Parents have different perceptions of bed-wetting than children from six to 15 years of age. Acta Paediatr. (2015) 104(10):e466–472. 10.1111/apa.13101 26119996

[3] Alanazi ANH Alanazi RSM Alanazi EN Alanazi RM Rabbani U. Prevalence of nocturnal enuresis among children and its association with the mental health of mothers in northern Saudi Arabia. Cureus. (2022) 14(2):e22232. 10.7759/cureus.22232 35340510 PMC8930502

[4] Hu HJ Zhang ZW Liang Y Luo YY Dou QF Song CP Prevalence, risk factors, and psychological effects of primary nocturnal enuresis in Chinese young adults. Int Neurourol J. (2021) 25(1):84–92. 10.5213/inj.2040398.149 33819961 PMC8022166

[5] Sousa E Silva GJ Sammour SNF Ferraro AA Koch VHK. Study of the profile of behavioral problems and quality of life indexes in a pediatric cohort of monosymptomatic enuresis. J Pediatr (Rio J). (2019) 95(2):188–93. 10.1016/j.jped.2017.12.006 29428322

[6] Walker RA. Nocturnal enuresis. Prim Care Clin Off Pract. (2019) 46(2):243–8. 10.1016/j.pop.2019.02.005 31030825

[7] Pedersen MJ Rittig S Jennum PJ Kamperis K. The role of sleep in the pathophysiology of nocturnal enuresis. Sleep Med Rev. (2020) 49:101228. 10.1016/j.smrv.2019.101228 31790860

[8] Soster LA Alves R Fagundes SN Koch VHK Bruni O. Sleep disturbances associated with sleep enuresis: a questionnaire study. Eur J Paediatr Neurol. (2016) 20(2):282–5. 10.1016/j.ejpn.2015.11.014 26732069

[9] Soster LA Fagundes SN Lebl A Alves RC Koch VH Spruyt K. Beyond bedwetting: how successful treatment is observed in sleep macrostructure. Sleep Med. (2024) 124:331–7. 10.1016/j.sleep.2024.09.035 39368160

[10] Song P Huang C Wang Y Wang Q Zhu W Yue Y Comparison of desmopressin, alarm, desmopressin plus alarm, and desmopressin plus anticholinergic agents in the management of paediatric monosymptomatic nocturnal enuresis: a network meta-analysis. BJU Int. (2019) 123(3):388–400. 10.1111/bju.14539 30216627

[11] Ring IJ Nevéus T Markström A Arnrup K Bazargani F. Nocturnal enuresis impaired children’s quality of life and friendships. Acta Paediatr. (2017) 106(5):806–11. 10.1111/apa.13787 28199734

[12] Rangel RA Seabra CR Ferrarez CEPF Soares JL Choi M Cotta RG Quality of life in enuretic children. Int Braz J Urol. (2021) 47(3):535–41. 10.1590/s1677-5538.ibju.2020.0308 33620999 PMC7993947

[13] Quiroz-Guerrero J Ortega-Pardo A Maldonado-Valadez RE De León RG-D Mercado-Villareal L Rodea-Montero ER. Maternal anxiety associated with nocturnal childhood enuresis. Children. (2022) 9(8):1232. 10.3390/children9081232 36010121 PMC9406453

[14] Tsai H-L Chang J-W Chen M-H Jeng M-J Yang L-Y Wu K-G. Associations between psychiatric disorders and enuresis in Taiwanese children: a national population-based study. Clin Epidemiol. (2020) 12:163–71. 10.2147/CLEP.S230537 32110107 PMC7035896

[15] Yilmaz EŞ Büyük ET. Effect of education given to children with enuresis on quality of life. J Pediatr Urol. (2021) 17(5):648.e1–e7. 10.1016/j.jpurol.2021.08.001 34518125

[16] Caldwell PH Codarini M Stewart F Hahn D Sureshkumar P. Alarm interventions for nocturnal enuresis in children. Cochrane db. Syst Rev. (2020) 2021(12):CD002911. 10.1002/14651858.CD002911.pub3 PMC7197139 32364251

[17] Huang H-M Wei J Sharma S Bao Y Li F Song J-W Prevalence and risk factors of nocturnal enuresis among children ages 5–12 years in Xi’an, China: a cross-sectional study. BMC Pediatr. (2020) 20(1):305. 10.1186/s12887-020-02202-w 32571248 PMC7310244

[18] Li W Yang G Tian W Li Y Zhang L Wang Y Bibliometric and visual analysis of nocturnal enuresis from 1982 to 2022. Front Pediatr. (2022) 10:972751. 10.3389/fped.2022.972751 36034562 PMC9412014

[19] Mohamed RA Youssef El-Sheikh O Noaman A. Applying health education learning package for mothers regarding nocturnal enuresis. Am J Nurs Res. (2019) 7(4):561–73. 10.12691/ajnr-7-4-18

[20] Iscan B Ozkayın N. Evaluation of health-related quality of life and affecting factors in child with enuresis. J Pediatr Urol. (2020) 16(2):195.e1–e7. 10.1016/j.jpurol.2019.12.018 32008988

[21] Klein MO Thews A Schulz-Jürgensen S. Health-related quality of life of children and adolescents with primary nocturnal enuresis who are undergoing therapy. Urologe. (2021) 60(9):1175–83. 10.1007/s00120-021-01549-x 34100127

[22] Shafiek H Evangelisti M Abd-elwahab NH Barreto M Villa MP Mahmoud MI. Obstructive sleep apnea in school-aged children presented with nocturnal enuresis. Lung. (2020) 198(1):187–94. 10.1007/s00408-019-00304-6 31828515

[23] Tai TT Tai BT Chang Y-J Huang K-H. The importance of understanding parental perception when treating primary nocturnal enuresis: a topic review and an institutional experience. Res Rep Urol. (2021) 13:679–90. 10.2147/RRU.S323926 34522688 PMC8434936

[24] Oztorun ZY Beser NG Oztorun K Arabacı LB. Evaluating the social anxiety depression levels and accompanying psychosocial problems in children diagnosed with enuresis. Cureus. (2022) 14(8):e28351. 10.7759/cureus.28351 36168363 PMC9506857

[25] Eray Ş Tekcan D Baran Y. More anxious or more shy? Examining the social anxiety levels of adolescents with primary enuresis nocturna: a controlled study. J Pediatr Urol. (2019) 15(4):343.e1–e5. 10.1016/j.jpurol.2019.04.002 31036479

[26] Durmaz O Kemer S Mutluer T Bütün E. Psychiatric dimensions in mothers of children with primary nocturnal enuresis: a controlled study. J Pediatr Urol. (2017) 13(1):62.e1–e6. 10.1016/j.jpurol.2016.06.018 27665376

[27] Al-Zaben FN Sehlo MG. Punishment for bedwetting is associated with child depression and reduced quality of life. Child Abuse Neglect. (2015) 43:22–9. 10.1016/j.chiabu.2014.11.007 25435105

[28] Ferrara P Franceschini G Di Castelbianco FB Bombace R Villani A Corsello G. Epidemiology of enuresis: a large number of children at risk of low regard. Ital J Pediatr. (2020) 46(1):128. 10.1186/s13052-020-00896-3 32917238 PMC7488742

[29] Berry AK. Helping children with nocturnal enuresis: the wait-and-see approach may not be in anyone’s best interest. AJN Am J Nurs. (2006) 106(8):56–63. 10.1097/00000446-200608000-00024 16905935

[30] Assimamaw NT Kebede AK Genetu KB. Effects of sex, toilet training, stress, and caffeine on nocturnal enuresis among school children in gondar town, the metropolitan city of Ethiopia: a community-based study in 2023. Front Pediatr. (2024) 12:1366430. 10.3389/fped.2024.1366430 38915871 PMC11194331

[31] The KIDSCREEN group C Michel G Bisegger C Fuhr DC Abel T. Age and gender differences in health-related quality of life of children and adolescents in Europe: a multilevel analysis. Qual Life Res. (2009) 18(9):1147–57. 10.1007/s11136-009-9538-3 19774493

[32] Kilicoglu AG Mutlu C Bahali MK Adaletli H Gunes H Metin Duman H Impact of enuresis Nocturna on health-related quality of life in children and their mothers. J Pediatr Urol. (2014) 10(6):1261–6. 10.1016/j.jpurol.2014.07.005 25164391

[33] Van Herzeele C Dhondt K Roels SP Raes A Groen L-A Hoebeke P Neuropsychological functioning related to specific characteristics of nocturnal enuresis. J Pediatr Urol. (2015) 11(4):208.e1–e6. 10.1016/j.jpurol.2015.04.033 26206411

[34] Kanaheswari Y Poulsaeman V Chandran V. Self-esteem in 6- to 16-year-olds with monosymptomatic nocturnal enuresis. J Paediatr Child Health. (2012) 48(10):E178–82. 10.1111/j.1440-1754.2012.02577.x 22998162

[35] Osman ZHH Ali SAO Kamel NMF. Impact of an educational program on mothers’ knowledge, attitude and practice regarding their children with nocturnal enuresis. Int J Adv Res. (2016) 4(6):771–82. 10.21474/IJAR01/723

[36] Elbahnasawy TH Elnagar AM. Psychological impact of nocturnal enuresis on self-esteem of school children. Am J Nurs Res. (2015) 3(1):14–20. 10.12691/ajnr-3-1-4

[37] Roccella M Smirni D Smirni P Precenzano F Operto FF Lanzara V Parental stress and parental ratings of behavioral problems of enuretic children. Front Neurol. (2019) 10:1054. 10.3389/fneur.2019.01054 31681143 PMC6797845

[38] Schlomer B Rodriguez E Weiss D Copp H. Parental beliefs about nocturnal enuresis causes, treatments, and the need to seek professional medical care. J Pediatr Urol. (2013) 9(6):1043–8. 10.1016/j.jpurol.2013.02.013 23608323 PMC4648250

[39] Sá CA De Souza SAM Villela MCBVA Souza VDM De Souza MHDF De Figueiredo AA Psychological intervention with parents improves treatment results and reduces punishment in children with enuresis: a randomized clinical trial. J Urol. (2021) 205(2):570–6. 10.1097/JU.0000000000001351 32924749

[40] Obe RL Stillman-Lowe C. Health education. Br Dent J. (2024) 236(3):181–5. 10.1038/s41415-024-7052-1 38332077

[41] Urlings J Sezer S Ter Laan M Bartels R Maal T Boogaarts J The role and effectiveness of augmented reality in patient education: a systematic review of the literature. Patient Educ Couns. (2022) 105(7):1917–27. 10.1016/j.pec.2022.03.005 35341611

[42] Wojtara MS. Use of social Media for patient education in dermatology: narrative review. JMIR Dermatol. (2023) 6:e42609. 10.2196/42609 37632938 PMC10335153

[43] Martínez-Miranda P Jiménez-Rejano JJ Rosales-Tristancho A Casuso-Holgado MJ. Comparative effect of different patient education modalities on quality of life in breast cancer survivors: a systematic review and network meta-analysis. Eur J Oncol Nurs. (2023) 67:102411. 10.1016/j.ejon.2023.102411 37806151

[44] Herval ÁM Oliveira DPD Gomes VE Vargas AMD. Health education strategies targeting maternal and child health: a scoping review of educational methodologies. Medicine (Baltimore). (2019) 98(26):e16174. 10.1097/MD.0000000000016174 31261550 PMC6616517

[45] Nishizaki N Hirano D Oishi K Shimizu T. YouTube videos in Japanese as a source of information on nocturnal enuresis: a content-quality and reliability analysis. Pediatr Int. (2022) 64(1):e15049. 10.1111/ped.15049 34747553

[46] Toprak T Tokat E. A quality analysis of nocturnal enuresis videos on YouTube. J Pediatr Urol. (2021) 17(4):449.e1–e6. 10.1016/j.jpurol.2021.03.014 33824069

[47] Bruno RR Wolff G Wernly B Masyuk M Piayda K Leaver S Virtual and augmented reality in critical care medicine: the patient’s, clinician’s, and researcher’s perspective. Crit Care. (2022) 26(1):326. 10.1186/s13054-022-04202-x 36284350 PMC9593998

[48] Addab S Hamdy R Thorstad K Le May S Tsimicalis A. Use of virtual reality in managing paediatric procedural pain and anxiety: an integrative literature review. J Clin Nurs. (2022) 31(21–22):3032–59. 10.1111/jocn.16217 35068011

[49] Chen F-Q Leng Y-F Ge J-F Wang D-W Li C Chen B Effectiveness of virtual reality in nursing education: meta-analysis. J Med Internet Res. (2020) 22(9):e18290. 10.2196/18290 32930664 PMC7525398

[50] Hermans ANL Betz K Verhaert DVM Den Uijl DW Clerx K Debie L 360° Virtual reality to improve patient education and reduce anxiety towards atrial fibrillation ablation. EP Eur. (2023) 25(3):855–62. 10.1093/europace/euac246 PMC10062331 36738261

[51] Siroosbakht S Rezakhaniha B. Is renal bladder ultrasound necessary in monosymptomatic primary nocturnal enuresis? A Case Control Study J Compr Pediatr. (2018) 9(4):e69006. 10.5812/compreped.69006

[52] Netto JMB Rondon AV Lima GRMD Zerati Filho M Schneider-Monteiro ED Molina CAF Brazilian consensus in enuresis-recomendations for clinical practice. Int Braz J Urol. (2019) 45(5):889–900. 10.1590/s1677-5538.ibju.2019.0080 31408290 PMC6844333

[53] Rezakhaniha S Rezakhaniha B Siroosbakht S. Limited caffeine consumption as first-line treatment in managing primary monosymptomatic enuresis in children: how effective is it? A randomised clinical trial BMJ Paediatr Open. (2023) 7(1):e001899. 10.1136/bmjpo-2023-001899 37072339 PMC10124248

[54] Gabr AA Gad MA Shalaby A. Quality of life in children with pseudoincontinence after implementing a bowel management program in Egypt. J Pediatr Surg. (2020) 55(2):261–4. 10.1016/j.jpedsurg.2019.10.048 31918852

[55] Palma PL Marzuillo P Di Sessa A Guarino S Capalbo D Marrapodi MM From clinical scenarios to the management of lower urinary tract symptoms in children: a focus for the general pediatrician. Healthcare. (2023) 11(9):1285. 10.3390/healthcare11091285 37174827 PMC10177757

[56] Nevéus T Fonseca E Franco I Kawauchi A Kovacevic L Nieuwhof-Leppink A Management and treatment of nocturnal enuresis-an updated standardization document from the international children’s continence society. J Pediatr Urol. (2020) 16(1):10–9. 10.1016/j.jpurol.2019.12.020 32278657

[57] Sajith S Patnaik SK Kanitkar M. Comparison of a voiding diary with clinical management tool as an outpatient screening tool for childhood functional voiding disorders. Indian Pediatr. (2021) 58(12):1147–50. 10.1007/s13312-021-2397-x 34183463

[58] Kwak KW Park KH. Clinical inconsistency of lower urinary tract symptoms between questionnaire and bladder diary in children with nocturnal enuresis. J Urol. (2008) 180(3):1085–90. 10.1016/j.juro.2008.05.053 18639291

[59] Jørgensen CS Kamperis K Walle JV Rittig S Raes A Dossche L. The efficacy of standard urotherapy in the treatment of nocturnal enuresis in children: a systematic review. J Pediatr Urol. (2023) 19(2):163–72. 10.1016/j.jpurol.2022.12.011 36641240

[60] Chan IH Wong KK. Common urological problems in children: primary nocturnal enuresis. Hong Kong Med J. (2019) 25(4):305–11. 10.12809/hkmj197916 31395789

[61] Larsson J Borgström M Karanikas B Nevéus T. Can enuresis alarm therapy be managed by the families without the support of a nurse? A prospective study of a real-world sample. Acta Paediatr. (2023) 112(3):537–42. 10.1111/apa.16634 36527281 PMC10107766

[62] Önol FF Guzel R Tahra A Kaya C Boylu U. Comparison of long-term efficacy of desmopressin lyophilisate and enuretic alarm for monosymptomatic enuresis and assessment of predictive factors for success: a randomized prospective trial. J Urol. (2015) 193(2):655–61. 10.1016/j.juro.2014.08.088 25158273

[63] Keten T Aslan Y Balci M Erkan A Senel C Oguz U Comparison of the efficacy of desmopressin fast-melting formulation and enuretic alarm in the treatment of monosymptomatic nocturnal enuresis. J Pediatr Urol. (2020) 16(5):645.e1–e7. 10.1016/j.jpurol.2020.07.018 32826183

[64] Alloussi SH Mürtz G Lang C Madersbacher H Strugala G Seibold J Desmopressin treatment regimens in monosymptomatic and nonmonosymptomatic enuresis: a review from a clinical perspective. J Pediatr Urol. (2011) 7(1):10–20. 10.1016/j.jpurol.2010.04.014 20576470

[65] Radojicic Z Milivojevic S Koricanac I Lazovic JM Laketic D Radojicic O Low compliance contribute to insufficient desmopressin response of primary monosymptomatic nocturnal enuresis and the role of voiding school. BMC Pediatr. (2021) 21(1):244. 10.1186/s12887-021-02714-z 34016082 PMC8136157

[66] Gasthuys E Dossche L Michelet R Nørgaard JP Devreese M Croubels S Pediatric pharmacology of desmopressin in children with enuresis: a comprehensive review. Pediatr Drugs. (2020) 22(4):369–83. 10.1007/s40272-020-00401-7 32507959

[67] Robson WLM Leung AKC Norgaard JP. The comparative safety of oral versus intranasal desmopressin for the treatment of children with nocturnal enuresis. J Urol. (2007) 178(1):24–30. 10.1016/j.juro.2007.03.015 17574054

[68] Gözüküçük A Kılıç M Çakıroğlu B. Desmopressin versus desmopressin+oxybutynin in the treatment of children with nocturnal enuresis. J Pediatr Urol. (2021) 17(4):451.e1–e6. 10.1016/j.jpurol.2021.04.001 33931318

[69] Issi Y Biçakci U. Does desmopressin withdrawal strategy affect relapse rates in monosymptomatic enuresis treatment? Eur J Pediatr. (2021) 180(5):1453–7. 10.1007/s00431-020-03918-8 33389072

[70] Thiagamoorthy G Cardozo L Robinson D. Current and future pharmacotherapy for treating overactive bladder. Expert Opin Pharmacother. (2016) 17(10):1317–25. 10.1080/14656566.2016.1186645 27253972

[71] Ghasemi K Esteghamati M Mohammadzadeh M Zare S. Desmopressin versus oxybutynin for nocturnal enuresis in children in bandar abbas: a randomized clinical trial. Electron Physician. (2016) 8(3):2187–93. 10.19082/2187 27123229 PMC4844487

[72] Chua ME Silangcruz JMA Chang S-J Yang SS. Immediate 1-month efficacy of desmopressin and anticholinergic combination therapy versus desmopressin monotherapy in the treatment of pediatric enuresis: a meta-analysis. J Pediatr Urol. (2016) 12(3):156.e1–e9. 10.1016/j.jpurol.2015.12.011 26922714

[73] Meyer SIR Jørgensen CS Kamperis K Andersen RF Pedersen MJ Faerch M Efficacy and safety of multimodal treatment in nocturnal enuresis - A retrospective cohort study. J Pediatr Urol. (2021) 17(4):447.e1–e7. 10.1016/j.jpurol.2021.03.005 33820712

[74] Esteghamati M Mousavi SE Zoghi G. Desmopressin plus tolterodine vs desmopressin plus indomethacin for refractory pediatric enuresis: an open-label randomized controlled trial. Indian Pediatr. (2023) 60(6):447–51. 10.1007/s13312-023-2906-1 37078485

[75] Dell’Osso B Palazzo MC Oldani L Altamura AC. The noradrenergic action in antidepressant treatments: pharmacological and clinical aspects. CNS Neurosci Ther. (2011) 17(6):723–32. 10.1111/j.1755-5949.2010.00217.x 21155988 PMC6493872

[76] Mello MF Locali RF Araujo RM Reis JN Saiovici S Mello LF A prospective and randomized study comparing the use of alarms, desmopressin and imipramine in the treatment of monosymptomatic nocturnal enuresis. J Pediatr Urol. (2023) 19(3):241–6. 10.1016/j.jpurol.2023.01.004 36717289

[77] Wang T-M Yang SS-D Tsai J-D Yu M-C Chiou Y-H Chen K-L Management of nocturnal enuresis in Taiwan: consensus statements of the Taiwan enuresis expert committee. J Formos Med Assoc. (2019) 118(6):965–72. 10.1016/j.jfma.2018.04.014 29779924

[78] Wenguang W Huifang Z Guiwen W Zhentie Y Rui H. Effect of traditional Chinese medicine zhiyi decoction and acupuncture on Serum ADH & inflammatory factors in patients of enuresis. Pak J Pharm Sci. (2019) 32(1(Special)):465–9. 30852486

[79] Ma Y Liu X Shen Y. Effect of traditional Chinese and western medicine on nocturnal enuresis in children and indicators of treatment success: randomized controlled trial. Pediatr Int. (2017) 59(11):1183–8. 10.1111/ped.13417 28891253

[80] Li J Hui X Yao L Shi A Yan P Yao Y The relationship of publication language, study population, risk of bias, and treatment effects in acupuncture related systematic reviews: a meta-epidemiologic study. BMC Med Res Methodol. (2023) 23(1):96. 10.1186/s12874-023-01904-w 37081403 PMC10120256

[81] Kannan P Bello UM. The efficacy of different forms of acupuncture for the treatment of nocturnal enuresis in children: a systematic review and meta-analysis. Explore. (2022) 18(4):488–97. 10.1016/j.explore.2021.11.008 34893441

[82] Radvanska E Kamperis K Kleif A Kovács L Rittig S. Effect of Laser acupuncture for monosymptomatic nocturnal enuresis on bladder reservoir function and nocturnal urine output. J Urol. (2011) 185(5):1857–62. 10.1016/j.juro.2010.12.068 21420107

[83] Karaman MI Koca O Küçük EV Öztürk M Güneş M Kaya C. Laser acupuncture therapy for primary monosymptomatic nocturnal enuresis. J Urol. (2011) 185(5):1852–6. 10.1016/j.juro.2010.12.071 21420121

[84] Radmayr C Schlager A Studen M Bartsch G. Prospective randomized trial using Laser acupuncture versus desmopressin in the treatment of nocturnal enuresis. Eur Urol. (2001) 40(2):201–5. 10.1159/000049773 11528199

[85] Alsharnoubi J Sabbour AA Shoukry AI Abdelazeem AM. Nocturnal enuresis in children between Laser acupuncture and medical treatment: a comparative study. Laser Med Sci. (2017) 32(1):95–9. 10.1007/s10103-016-2090-9 27744492

[86] Liu M Li Y Xian J Yang W Gao Q Yu J. Pediatric tuina (massage) for primary monosymptomatic nocturnal enuresis: a protocol for systematic review and meta-analysis. Medicine (Baltimore). (2020) 99(51):e23738. 10.1097/MD.0000000000023738 33371128 PMC7748190

[87] Van Poecke AJ Cunliffe C. Chiropractic treatment for primary nocturnal enuresis: a case series of 33 consecutive patients. J Manipulative Physiol Ther. (2009) 32(8):675–81. 10.1016/j.jmpt.2009.08.019 19836605

[88] Kuru K Ansell D Jones M De Goede C Leather P. Feasibility study of intelligent autonomous determination of the bladder voiding need to treat bedwetting using ultrasound and smartphone ML techniques: intelligent autonomous treatment of bedwetting. Med Biol Eng Comput. (2019) 57(5):1079–97. 10.1007/s11517-018-1942-9 30588575 PMC6477014

[89] Kuru K Ansell D Jones M Watkinson BJ Caswell N Leather P Intelligent autonomous treatment of bedwetting using non-invasive wearable advanced mechatronics systems and MEMS sensors: intelligent autonomous bladder monitoring to treat NE. Med Biol Eng Comput. (2020) 58(5):943–65. 10.1007/s11517-019-02091-x 32090271 PMC7188739

[90] Caswell N Kuru K Ansell D Jones MJ Watkinson BJ Leather P Patient engagement in medical device design: refining the essential attributes of a wearable, Pre-void, ultrasound alarm for nocturnal enuresis. Pharmaceut Med. (2020) 34(1):39–48. 10.1007/s40290-019-00324-w 31970684

[91] Kuru K Ansell D Hughes D Watkinson BJ Gaudenzi F Jones M Treatment of nocturnal enuresis using miniaturised smart mechatronics with artificial intelligence. IEEE J Transl Eng Health Med. (2024) 12:204–14. 10.1109/JTEHM.2023.3336889 38088989 PMC10712671

